# Transferability of MACE Graph Neural Network for Range Corrected Δ‑Machine Learning Potential QM/MM Applications

**DOI:** 10.1021/acs.jpcb.5c02006

**Published:** 2025-05-26

**Authors:** Timothy J. Giese, Jinzhe Zeng, Darrin M. York

**Affiliations:** † Laboratory for Biomolecular Simulation Research, Institute for Quantitative Biomedicine, and Department of Chemistry and Chemical Biology, Rutgers University, Piscataway 08854, New Jersey, United States; ‡ School of Artificial Intelligence and Data Science, University of Science and Technology of China, Hefei 230026, China; § 12652 Suzhou Institute for Advanced Research, University of Science and Technology of China, Suzhou Big Data & AI Research and Engineering Center, Suzhou 215123, China

## Abstract

We previously introduced a “range corrected” Δ−machine learning potential (ΔMLP) that used deep neural networks to improve the accuracy of combined quantum mechanical/molecular mechanical (QM/MM) simulations by correcting both the internal QM and QM/MM interaction energies and forces [J. Chem. Theory Comput. 2021, 17, 6993–7009]. The present work extends this approach to include graph neural networks. Specifically, the approach is applied to the MACE message passing neural network architecture, and a series of AM1/d + MACE models are trained to reproduce PBE0/6–31G* QM/MM energies and forces of model phosphoryl transesterification reactions. Several models are designed to test the transferability of AM1/d + MACE by varying the amount of training data and calculating free energy surfaces of reactions that were not included in the parameter refinement. The transferability is compared to AM1/d + DP models that use the DeepPot-SE (DP) deep neural network architecture. The AM1/d + MACE models are found to reproduce the target free energy surfaces even in instances where the AM1/d + DP models exhibit inaccuracies. We train “end-state” models that include data only from the reactant and product states of the 6 reactions. Unlike the uncorrected AM1/d profiles, the AM1/d + MACE method correctly reproduces a stable pentacoordinated phosphorus intermediate even though the training did not include structures with a similar bonding pattern. Furthermore, the message passing mechanism hyperparameters defining the MACE network are varied to explore their effect on the model’s accuracy and performance. The AM1/d + MACE simulations are 28% slower than AM1/d QM/MM when the ΔMLP correction is performed on a graphics processing unit. Our results suggest that the MACE architecture may lead to ΔMLP models with improved transferability.

## Graphical abstract

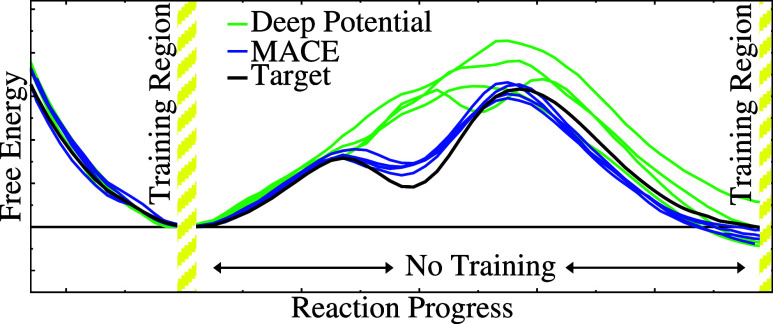

## 1 Introduction

Simulation of biochemical reactions is challenging due to the broad range of spatial and temporal scales involved.^1–3^ Often the goal of these simulations is to identify the minimum free energy pathway that defines the mechanism and determines the reaction rate.^4,5^ This typically requires extensive sampling of high-dimensional free energy surfaces^6^ and the use of efficient free energy path methods.^7^ In order to model chemical bond formation and cleavage processes, a quantum mechanical/molecular mechanical (QM/MM) potential may be used.^8,9^ Although good accuracy can be obtained with ab initio density-functional models and a reliable basis set, their evaluation is computationally intensive. The large computational cost places practical restrictions on the number of atoms that can be treated quantum mechanically and/or the amount of sampling that can be reasonably afforded. These restrictions, in turn, place limitation on the predictive capabilities of the method.

Machine learning potentials (MLP) have emerged as a cost-effective alternative to expensive ab initio evaluation.^10–17^ Pure MLPs abandon physics-based mathematical modeling in favor of neural network calculation of the total potential energy.^18^ Although pure MLPs can be trained to reproduce target data within the scope of their parametrization,^19,20^ they lack a treatment for long-range electrostatic interactions which are critical to correctly simulate the condensed phase, macromolecular systems, and interfacial properties.^21–24^ This has led to the development of long-range corrections to supplement pure MLPs.^25–30^ The so-called ΔMLP approach uses the opposite strategy: the MLP is a correction to (as opposed to a replacement for) an inexpensive physics-based model.^31–37^ The long-range interactions are calculated by the inexpensive base model, and the MLP is a short-range correction. Therefore, the ΔMLP approach can be easily adapted for use with semiempirical quantum mechanical/molecular mechanical (QM/MM) calculations with electrostatic^33,34,38–41^ or mechanical embedding.^42^ Typically, the ΔMLP is parametrized to correct semiempirical QM/MM energies and forces to match ab initio QM/MM target data; however, some methods are trained to reproduce target data that also includes polarization of the nearby MM surrounding.^33,43^ Several QM/MM ΔMLP strategies use neural networks to explicitly correct the internal QM interactions in a manner that implicitly accounts for the MM environment.^38,44–48^ In contrast, Böselt et al.^49^ and Zeng et al.^34^ independently proposed “range corrected” extensions of the QM/MM ΔMLP strategy that use neural networks to explicitly correct the interactions between QM and nearby MM atoms in a manner that yields smooth potential energies as MM atoms drift into (or away from) the vicinity of the QM region during the course of simulation.

Applications of the range corrected ΔMLP QM/MM strategy^34,39–41,50–56^ were aided by the availability of an interface between the DeePMD-kit software and sander molecular dynamics program^51,52,57–59^ available as part of AmberTools.^60^ The ΔMLPs in those works were limited to using the DeepPot-SE (DP) deep neural network potential implemented with the TensorFlow libraries.^61^ Many of the new MLPs appearing in the literature, however, are graph neural networks (GNNs) developed with PyTorch.^62^ The publication of new neural network models are often accompanied by software that implements the method and provides basic infrastructure for training the network parameters. The differences between the ad hoc software infrastructure introduces an obstacle for consistently training different models with the same optimization algorithms, hyperparameters, and active learning strategy. The comparisons are further inconvenienced by needing to interface each method to the molecular dynamics program. We have recently extended the DeePMD-kit software with a software plugin architecture, designated DeePMD-GNN,^59,63^ that allows it to evaluate graph neural networks developed with PyTorch.^62^ The plugin architecture to DeePMD-kit causes the new MLPs to immediately become available within sander—or any dynamics program interfaced with DeePMD-kit.^57,59^ Furthermore, the various MLPs can be trained with the aid of the DP-GEN software so that they may be compared using a consistent set of training algorithms.^64^

In the present work, we describe a new range corrected ΔMLP based on the MACE message passing neural network architecture.^65,66^ The range corrected graph topology is designed to improve the accuracy of QM and nearby QM/MM interactions within a cutoff. We parametrize a series of ΔMLPs that correct AM1/d^67,68^ QM/MM to reproduce PBE0/6–31G* QM/MM target energies and forces. The accuracy of MACE and DP ΔMLPs are tested by comparing their ability to reproduce the free energy surfaces of 6 nonenzymatic phosphoryl transesterification reactions shown in Figure 1. The comparisons investigate the transferability of the MACE and DP architectures as one varies the amount of training data. Furthermore, we explore their transferability to reactions not included in the parametrization. The quality of the models is judged by their ability to reproduce a reference free energy surface and by their ability to be used as a reference potential to estimate the high-level surface from reweighted AM1/d ΔMLP sampling. Finally, we train a series of MACE ΔMLPs with various network hyperparameters to quantify their effect on the model’s performance and accuracy.

**Figure 1:**
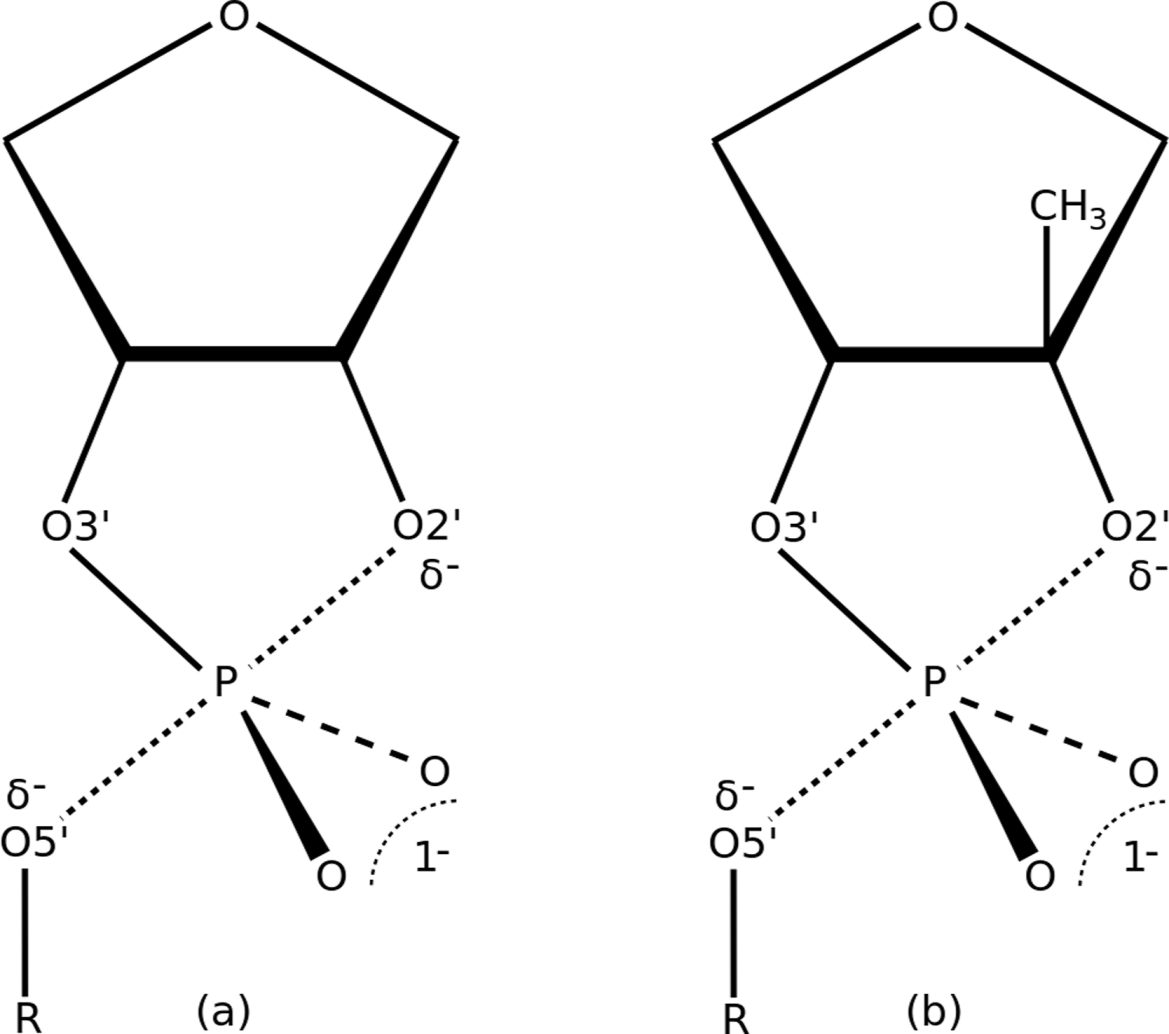
Nonenzymatic phosphate transesterification reactions examined in this work. Parts a and b illustrate the reaction with the nucleophile (Nuc) and C2′-methylated nucleophile (mNuc), respectively, where the atom labels use the ribonucleic acid numbering convention. The reactant state has a fully formed O5′-P bond and a broken P–O2′ bond. The product state has a P–O2′ covalent bond and a broken O5′-P bond. The leaving groups (RO5′) considered in this work are ethoxide (EtO), acetate (AcO), and phenoxide (PhO).

The nucleophile (Nuc) and methylated nucleophile (mNuc) shown in Figure 1a,b, respectively, are hydroxyalkyl phosphate esters that undergo phosphoryl transesterification with either an ethoxide (EtO), acetate (AcO), or phenoxide (PhO) leaving group. These are nonenzymatic models for the RNA-cleavage reactions^69^ occurring in various nucleolytic ribozymes,^70^ including hammerhead,^71–75^ hairpin,^76–79^ HDV,^80–82^ VS,^83–85^ *glmS*,^86,87^ twister,^88–91^ pistol,^92–96^ TS,^93,97,98^ and hatchet^93^ ribozymes. Under physiological conditions, the RNA transphosphorylation mechanism includes: deprotonation of O2′ by a general base, nucleophilic attack of the scissile phosphate by the activated O2′, departure of the O5′ leaving group, and protonation of the O5′ by a general acid. In contrast, the model reactions explored in the present work are performed in basic conditions, in which case the O2′ and O5′ remain deprotonated throughout the reaction. The reaction progress is therefore characterized by the phosphoryl transfer reaction coordinate $\xi =\left|R_{{O5}^{\prime }}-R_{P}\right|-\left|R_{P}-R_{{O2}^{\prime }}\right|$ . The mNuc nucleophile shown in Figure 1 introduces a methyl group at the C2′ position to make the O2′ a secondary alkoxide, like what is found in the native enzymatic reactions. A similar series of model reactions have been studied using linear free energy relationships,^99,100^ and it was found that the mechanism is correlated to the leaving group p*K*_a_. The reaction was found to proceed through a concerted mechanism containing a single, early (ξ < 0) transition state when the leaving group p*K*_a_ is less than 11. Alternatively, the reaction proceeds through an associative mechanism when the leaving group p*K*_a_ is greater than 12. The associative mechanism contains two barriers separated by a minimum. The early transition state corresponds to partial formation of a P–O2′ bond, and the rate controlling (late) transition state is characterized by partial cleavage of the P–O5′ bond. The p*K*_a_ values of the leaving groups examined in this work are 15.5 (EtO), 4.76 (AcO), and 9.89 (PhO); therefore, one would expect only the Nuc-EtO and mNuc-EtO reactions to display a pentacoordinated phosphorus intermediate and a late rate-controlling transition state.

The remainder of the paper is organized as follows. The Methods section summarizes the main differences between the DP and MACE architectures, and we describe the necessary changes to the MACE graph topology that are required to make the architecture suitable for use as a ΔMLP in QM/MM applications. The computational details of the umbrella sampling used to calculate the free energy surfaces are provided. The AM1/d QM/MM sampling was performed to obtain free energy surfaces, and various subsets of the saved samples were used to train the ΔMLPs. The training strategy is described and the optimization hyperparameters are provided. The Results and Discussion section begins by demonstrating that the PBE0/6–31G* free energy surfaces cannot be reliably estimated by reweighting the AM1/d QM/MM sampling. The transferability of the DP and MACE models are compared by training them to 3 of the 6 reactions. The models are used in umbrella sampling to predict the free energy surfaces of all 6 reactions. It is shown that the AM1/d + MACE potential reproduces all 6 surfaces, whereas the AM1/d + DP potential displays artifacts in the 3 untrained surfaces. We then compare DP and MACE models trained only to the reactant and product states without providing them training data between these states. It is shown that the error in the resulting AM1/d + MACE free energy surface are much smaller than the AM1/d + DP surfaces. The AM1/d + MACE samples also better agree with the reference ab initio free energy surface when the sampling is reweighted. Finally, we train a series of MACE models that vary the network hyperparameters to observe the relative effect of each parameter on the model’s accuracy and performance. The manuscript concludes with a summary of the results.

## 2 Methods

We recently reported interoperable software infrastructure for next-generation QM/MM-ΔMLP force fields.^51,52^ where the ΔMLP is a nonelectrostatic correction applied to the semiempirical QM and short-range QM/MM interactions and parametrized to reproduce an ab initio QM/MM method. The infrastructure was originally created to develop “range-corrected” DP models for semiempirical QM/MM applications.^34,39^ By “range-corrected”, we mean that the MLP modifies the short-range QM/MM interactions (in addition to the semiempirical QM energy) in a manner that avoids energy discontinuities as MM atoms move to and away from the vicity of the QM region. We recently extended the DeePMD-kit software to support GNNs,^59,63^ and present work describes how the graph’s network edges are assigned to adhere to the range-corrected QM/MM framework. The approach is demonstrated by developing QM/MM-ΔMLP potentials using the MACE message passing neural network architecture, and the transferability of the MACE ΔMLP is compared to DP models trainined to the same data. Below we describe the machine learning models, the simulation details, and the neural network training.

### 2.1 Machine Learning Potentials

The range corrected DP models use deep neural networks rather than graph neural networks to calculate the ΔMLP correction.^34,39^ The energy is decomposed into atomic components (site energies), and the contribution from each atom is the output of a fitting network composed of 3 hidden layers with 240 neurons per layer. The input to the fitting network is a “feature matrix” that is calculated from embedding and coordinate matrices. The embedding matrix uses the hybrid descriptor infrastructure within DeePMD-kit^57^ to define descriptors for the QM and QM/MM interactions. The present work uses the deep potential smooth edition descriptor with type embedding.^101,102^ The embedding matrices are calculated from deep neural networks composed of 3 layers consisting of 25, 50, and 100 neurons. Embedding networks are trained for each chemical species. The QM atom chemical species is assigned by atomic symbol (e.g., “H” for a QM hydrogen), whereas the MM atoms are assigned by the atomic symbol prefixed by the letter “m” (e.g., “mH” for a MM hydrogen). In this manner, QM and MM atoms of the same element can have different network parameters. The contribution of the MM atoms to the QM/MM “coordinate matrix” smoothly approaches 0 at a 6 Å cutoff, and the feature matrix includes 12 axis filters. The DP model’s atomic contribution to the energy includes a “bias”—a constant that persists when the atom is isolated. The bias of the MM atoms is forced to be zero to prevent discontinuous changes to the total energy as MM atoms diffuse to (or from) the QM region’s vicinity. The DP network described here is similar in size to ΔMLPs trained in previous works.^34,39,40,103^

The range corrected MACE models are message passing neural networks^65^—a type of graph neural network—where the nodes of the graph are the atoms and the edges define the topology of the communication pattern.^104^ In the original description of MACE, the edges consist of all atom pairs within a cutoff.^65^ The MACE energy does not encounter a discontinuity as an interatomic distance exceeds the cutoff because the distance features are embedded using Bessel basis functions and a polynomial envelope that smoothly forces the feature to zero. In contrast, the edges within the range corrected MACE ΔMLP include all atom pairs within a cutoff if at least one of the atoms in the pair is a QM atom. In other words, the edges connecting MM atoms to other MM atoms are excluded from the graph because the ΔMLP only corrects the QM and QM/MM interactions. The MACE total energy includes a contribution from each site corresponding to its isolated atomic energy. One often chooses the isolated site energies from a linear regression to a collection of molecular energies. For a ΔMLP, these are instead isolated atom energy corrections (the difference between the base and target levels of theory). The isolated MM atom energy corrections must be zero to ensure the range corrected MACE energy is continuous as a MM atom traverses its cutoff with the QM atoms. Figures S1 in the Supporting Information illustrates the smoothness of the QM and QM/MM MACE corrections, and Figure S2 demonstrates energy conservation in microcanonical QM/MM simulations using a AM1/d + MACE potential. Like the DP model, the inputs to the MACE network are the chemical species and atomic coordinates. The chemical species of the QM atoms are assigned by atomic number. We reserved a portion of the periodic table to assign chemical species for the MM atoms by adding 50 to their atomic number. This is the mechanism which allows a MM element’s network parameters to be trained differently than the corresponding QM element. From the perspective of MACE, the MM atoms are treated in the same manner as QM atoms; however, pairs of MM atoms do not contribute edges and the MM atomic energy bias is restricted to be zero.

A graph neural network has previously been used within a range corrected ΔMLP QM/MM model.^105^ In that work, the graph was described as consisting of nodes and edges that represent atoms and their interactions up to a cutoff radius, respectively. It was not explicitly articulated whether their graph structure excluded edges formed by MM pairs, if the MM energy biases were forced to zero, or if the MM atoms have different network parameters as corresponding QM elements; however, our interpretation of their work and previous models^49^ lead us to believe their QM/MM ΔMLP implementation is ostensibly similar to what we have presented. Aside from the details of the QM/MM implementation, the GNN used in ref 105 is significantly different than the MACE model employed in the present work, in part because GNN frameworks have matured at a rapid pace.^106^ Whereas ref 105 constructs 2-body messages (messages calculated from atomic pairs) from invariant features, the Atomic Cluster Expansion (ACE) framework proposed a many-body expansion to describe the local environment.^107^ The SphereNet GNN model similarly found it beneficial to construct 3-body messages; however, the computational performance suffered from explicit enumeration of triplets.^108^ Other GNNs, such as NequIP, were shown to improve accuracy by using equivariant internal features, but many message passing layers were required.^109^ The MACE model combines the ACE framework with equivariant internal features to build many-body messages.^65,66^ It was found to reduce the number of message passing layers needed to achieve comparable accuracy, and the computational efficiency is maintained by building high order features from tensor products of lower order features rather than explicit enumeration of *n*-tuplets. The maximum body order is controlled by a hyperparameter called the “correlation”, ν, which is the body order minus 1. Values of ν = 1 and ν = 2 would construct 2-body and 3-body features, respectively. The correlation does not refer to the “receptive field”, which is the set of nodes which ultimately influence the output of a given target node. The first layer of messages are constructed from a local environment, but their influence can extend beyond the cutoff as the messages are iteratively communicated through the graph. The number of message passing layers, *T*, is another hyperparameter.

Unless otherwise noted, the following hyperparameters were set to define the range corrected MACE models trained in this work. Radial features are generated using a 6 Å cutoff, 8 Bessel basis functions, and a polynomial envelope of order 5, which was fed to a 3-layer perceptron consisting of 64 neurons/layer. The angular description of the local environment is expanded in spherical harmonics to order 3. The equivariant message passing uses spherical harmonics up to order *L* = 1 with *N* = 128 embedding channels and a correlation order of ν = 3. The MLP consists of *T* = 2 message passing layers. The readout of the last layer is passed through a 16 channel perceptron and gated with the sigmoid linear unit function. In some instances, we make comparison to models that vary the values of *L*, ν, *T*, and *N* to observe their effect on accuracy and performance.

### 2.2 Computational Details

Simulations were carried out with recently developed software infrastructure in AmberTools^60^ for use with QM/MM-ΔMLP force fields.^51,52^ The 6 nonenzymatic reactions were prepared by solvating each system with 2200 TIP3P water molecules in a 40.8 Å cubic unit cell. The solute is the QM region (see Figure 1), and the solvent is the MM region. Hydrogen mass repartitioning was applied to the solute atoms to allow for stable QM/MM dynamics with a 2 fs time step.^110^ The system density was equilibrated with a MM potential in the isothermal–isobaric ensemble with the Langevin thermostat and Berendsen barostat^111^ for 1 ns at 298 K and 1 atm while using a 5 ps^–1^ collision frequency. The electrostatics were evaluated with the particle mesh Ewald method using 9 Å real-space cutoffs, a 1 Å reciprocal space grid, and tinfoil boundary conditions.^112^ The solute net charge was neutralized with a uniform background correction.^113,114^ The AM1/d QM/MM potential was used to extend the simulation for an additional 10 ps.^67,68^ The semiempirical QM/MM electrostatics were evaluated with the Mulliken charge particle mesh Ewald method.^8^ The umbrella sampling consisted of 64 values of ξ ranging from −3.3⁡Å≤ξ≤3.0⁡Å in increments of 0.1 Å. All harmonic potential force constants were set to 300 kcal mol^–1^ Å^–2^. An initial structure for each window was obtained by scanning the reaction coordinate in a sequential series of 2 ps *NVT* simulations departing from the reactant state. Each window was then independently equilibrated for 200 ps in the *NVT* ensemble at 298 K, followed by an additional 200 ps of production sampling. The positions and forces of 500 samples from each production simulation were saved. The PBE0/6–31G* QM/MM energies and forces of each saved sample was reevaluated for free energy analysis and ΔMLP training. The ab initio QM/MM electrostatics were evaluated with the ambient potential composite Ewald method.^115^

The training produces 4 ΔMLP models obtained from independent stochastic optimizations initiated from different random number seeds. AM1/d ΔMLP umbrella sampling was then performed with each model. The 64 windows were re-equilibrated with the AM1/d ΔMLP for 20 ps in the canonical ensemble at 298 K. This was followed by 50 ps of production sampling, from which 250 samples were saved. Free energy surfaces were calculated from the multistate Bennett acceptance ratio (MBAR) method and a 0.1 Å histogram bin spacing^116,117^ using the implementation in FE-ToolKit.^6^ The optimal choice of histogram bin width is dependent upon the underlying features of the surface. A detailed analysis of the histogram bin widths on analogous nonenzymatic phosphoryl transfer reaction surfaces has been presented elsewhere.^6^ It was found that bin widths smaller than 0.1 Å unnecessarily introduce numerical noise without signifcantly changing the activation free energy. Some have proposed using small histogram bins and postprocessing the noisy data with Gaussian Process Regression or other smoothing techniques.^117^ The curves illustrated in the current work have not been smoothed; the curves are a series of straight line segments that connect the free energy values at the bin centers. The potential energy of each sample was reevaluated with the 4 AM1/d ΔMLP models and the PBE0/6–31G* QM/MM reference. These energy evaluations were used to estimate the PBE0/6–31G* QM/MM free energy surface from the AM1/d ΔMLP sampling using either the weighted thermodynamic perturbation (wTP) method^117–121^ or the generalized weighted thermodynamic perturbation (gwTP) method.^40^ The wTP method reweights the samples obtained from a single reference potential. There are 4 reference potentials (the 4 AM1/d ΔMLP models) resulting in 4 wTP estimates. In contrast, the gwTP method reweights the sampling obtained from multiple reference potentials to produce a single estimate of the target free energy surface. If the reference and target potentials always agreed, then the observed distribution of samples would not need to be reweighted to mimic the target potential’s expected distribution. If the reference and target distributions poorly overlapped, then the reweighting becomes unreliable. The presence of poor overlap is often evident when the reweighting is dominated by a nonnegligble weight from only a few samples. One can quantify the weight distribution within each histogram bin from a quantity called the “reweighting entropy”.^122^ The reweighting entropy is a unitless number between 0 and 1. It is 1 when the weights are uniform, and it is close to zero when the nonzero weights are dominated by a few samples. Previous studies have found that reweighting methods become unreliable when the reweighting entropy drops below 0.6.^40,117^ The free energy surface calculations employed the density of states smoothing algorithm described in ref 118 using a 0.2 kT energy histogram. This algorithm dampens outlier weights within a spatial bin, as opposed to smoothing the free energy values between spatial bins. Nevertheless, it was found to reduce the numerical noise and slightly increase the reweighting entropy values.

### 2.3 Neural Network Training

The neural network training was performed with the “simplify” workflow within the DP-GEN software.^64^ In this workflow, one has a fixed database of samples that are partitioned into training and test subsets, and the neural network parameters are optimized using the Adam stochastic gradient descent method to reproduce the training data.^123^ The training is repeated 4 times using different random number seeds to produce 4 neural network parameter sets. The training subset is discarded and the 4 parameter sets are applied to the samples in the test set. If the maximum root-mean-square error of the atomic force vectors is less than 0.08 eV/Å, then the sample is discarded from the test set. The whole process repeats by treating the revised test set as the new database which is repartitioned into training and test subsets. The optimization is restarted with the new training set. The process terminates when all of the database samples have been exhausted. In the present work, the first training set contains 50% of the database samples selected at random. In the second and subsequent iterations, the training set contains no more than 25% of the original database size. In this manner, the process is guaranteed to terminate after 3 rounds of optimizations; however, it often finishes after 2 rounds because the first set of parameters is usually accurate enough to discard most of the test set.

Each Adam optimization was performed with an exponential learning rate that decays from 10^–3^ to 10^–5^ over the course of 400,000 steps. The loss function is a weighted sum of squared differences in the predicted and target energy and force corrections.^57^ The weight on the energy errors exponentially increase from 1 eV^–2^ to 100 eV^–2^ during the optimization whereas the weight on the force errors remain fixed at 100 Å^2^·eV^–2^.

The ΔMLPs generated in this work were prepared by first parametrizing an “end-state model”. The end-state models are trained to the AM1/d QM/MM sampling of the reactant and product states from all 6 reactions. Specifically, the training data consisted of the 500 samples saved from the 12 simulations corresponding to the ξ = – 2.0 Å and ξ = 3.0 Å states of the 6 reactions. The amount of training data was extended 10% by selecting 50 samples from each ensemble and making small, random displacements to the QM atomic positions. Each QM heavy atom was displaced by up to 0.15 Å in a random direction, and the each hydrogen was displaced along its covalent bond to change its length by a random amount in the range 0.7 Å to 1.2 Å. Every DP and MACE model was parametrized to the same end-state training data.

The end-state models can be directly used in production sampling to calculate free energy surfaces, or they can be used to restart the optimization to create a “fine–tuned” model that is trained to additional data. All of the ΔMLPs discussed in the present work are end-state models except those referred to as S:1, S:2, S:4, and S:8 whose training was restarted to include umbrella sampling from other ξ values. The S:1 ΔMLP includes all 64 windows (−3.3 Å ≤ ξ ≤ 3.0 Å in steps of 0.1 Å) from each of the mNuc/EtO, Nuc/AcO, and Nuc/PhO reactions. The S:2 ΔMLP includes 32 windows (−3.3 Å ≤ξ ≤ 2.9 Å in steps of 0.2 Å) from each of the mNuc-EtO, Nuc-AcO, and Nuc-PhO reactions. The S:4 ΔMLP includes 16 windows (−3.3 Å ≤ ξ ≤ 2.7 Å in steps of 0.4 Å) from each of the mNuc-EtO, Nuc-AcO, and Nuc-PhO reactions. The S:8 ΔMLP includes 8 windows (−3.3 Å ≤ ξ ≤ 2.3 Å in steps of 0.8 Å) from each of the mNuc-EtO, Nuc-AcO, and Nuc-PhO reactions. None of the fine–tuned models are provided additional data from the mNuc-AcO, mNuc-PhO, nor Nuc-EtO reactions. We will show that the S:1, S:2, S:4, and S:8 MACE ΔMLPs produce nearly indistinguishable gwTP estimates of the PBE0/6–31G* free energy surfaces. Furthermore, their reweighting entropies exceed 0.8, which suggest the estimates are reliable. The “reference” PBE0/6–31G* free energy surfaces appearing in the comparisons are the average of these 4 gwTP estimates. Each reference surface is produced from an aggregate of 76.8 ns of QM/MM sampling.

## 3 Results and Discussion

### 3.1 AM1/d QM/MM Free Energy Profiles

All of the ΔMLPs explored in the present work are corrections to a AM1/d QM/MM potential that are parametrized to reproduce PBE0/6–31G* QM/MM energies and forces. Figure 2 compares the AM1/d and PBE0/6–31G* reference free energy surfaces to appreciate the magnitude of the desired corrections. The AM1/d rate limiting transition states are 1 to 5 kcal/mol higher than PBE0/6–31G*, and the locations of the AM1/d reactant state minima are shifted by 0.4 Å in the + ξ direction. The PBE0/6–31G* mNuc-EtO and Nuc-EtO profiles exhibit two barriers separated by an intermediate, whereas the AM1/d profiles have only one barrier. Figure 2 also shows the PBE0/6–31G* surfaces estimated from wTP reweighting of the AM1/d sampling. The uncorrected AM1/d method is not an adequate reference potential; it produces target free energy surfaces that exhibit large amounts of numerical noise. The lack of phase space overlap between AM1/d and PBE0/6–31G* is also expressed by the presence of very low reweighting entropy values, which range from 0.12 to 0.24.

**Figure 2:**
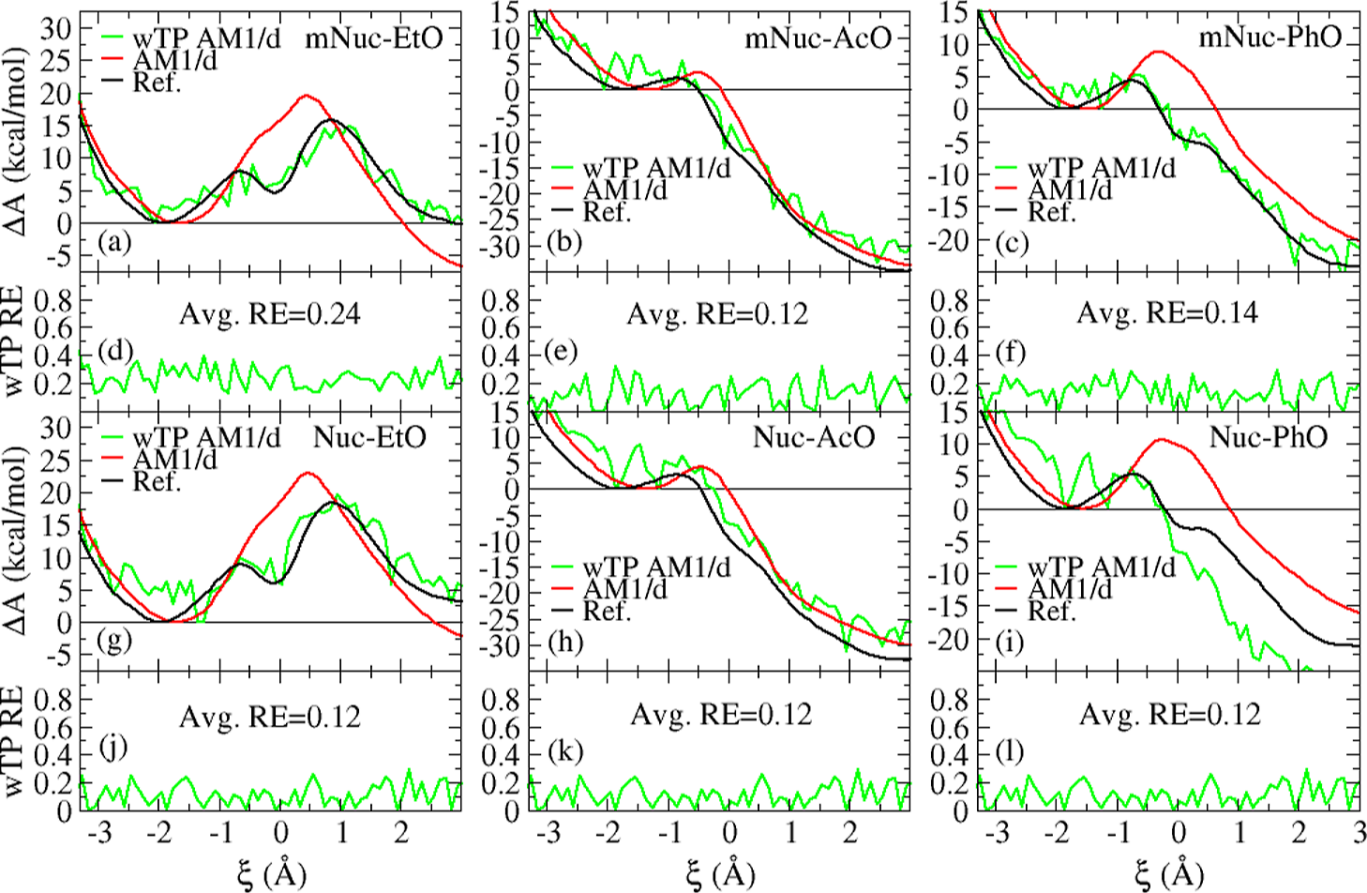
PBE0/6–31G* free energy profile estimated from wTP analysis (green line) of the AM1/d (red line) sampling. The reference curve is the average of the 4 gwTP estimates shown in Figure 3. Parts a–f and g–l are the reactions involving the mNuc and Nuc nucleophiles, respectively.

### 3.2 Comparison of DP and MACE Transferability

The sensitivity and transferability of MACE and DP ΔMLPs are examined in Figures 3 and 4, respectively. The models shown in these 2 figures refine the end-state parametrization by restarting the network optimization with umbrella sampling taken from the mNuc-EtO, Nuc-AcO, and Nuc-PhO systems. The training data includes umbrella windows extracted with a stride S:*n*, where *n* is the integer stride through the sequence of 64 windows. In other words, the S:8 models contain large gaps in the training data relative to the S:1 models. Sampling from the Nuc-EtO, mNuc-AcO, and mNuc-PhO reactions were not included in the model refinement.

**Figure 3:**
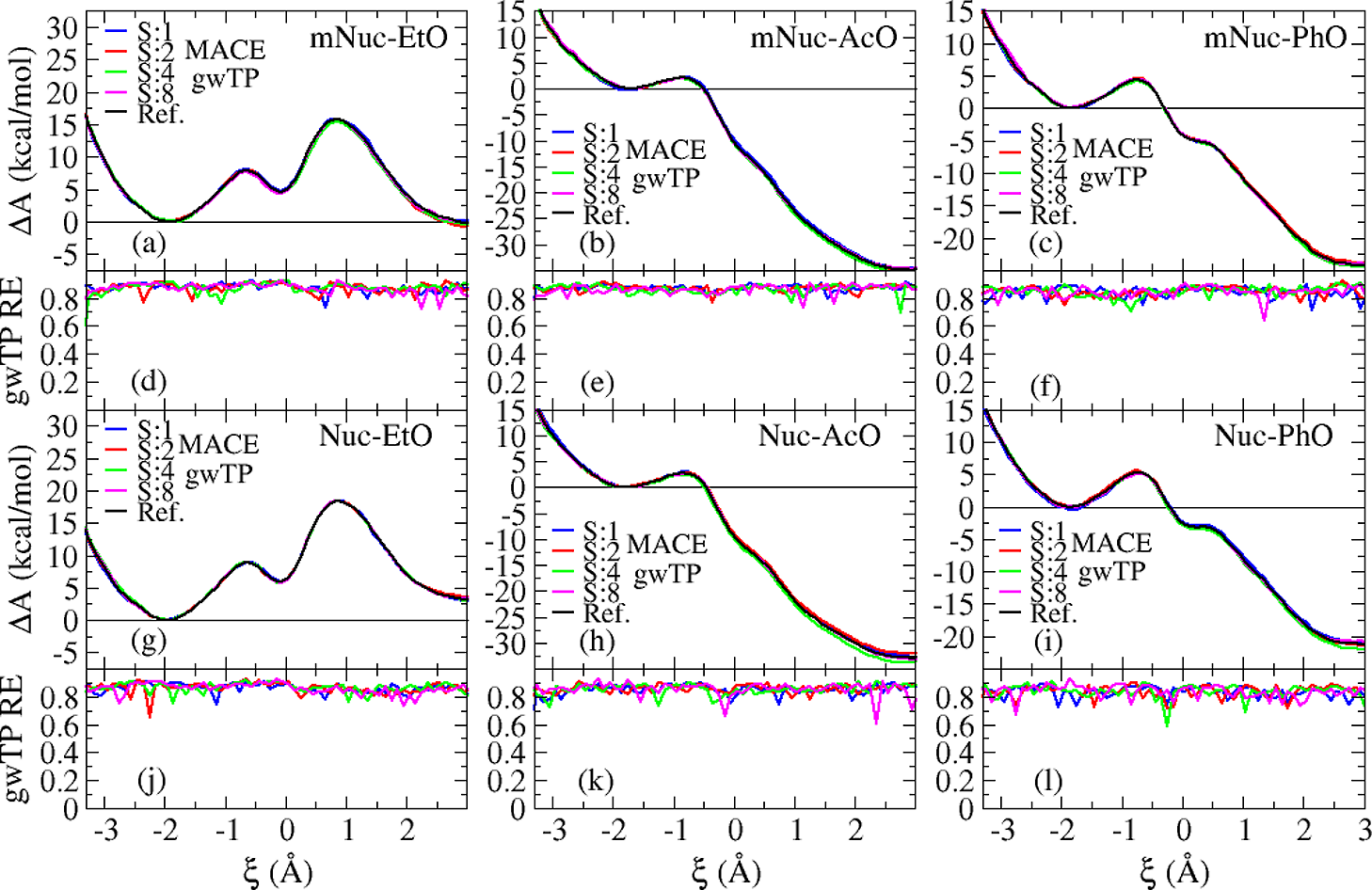
Estimates of the PBE0/6–31G* free energy profile made from gwTP analysis of several MACE parametrizations. The S:1 models were parametrized to the NCH–OEt, NHH-OAc, and NHH-OPh reactions using all 64 umbrella windows. The other S/*n* models were parametrized with fewer samples. *n* is the stride in the ξ series of umbrella windows used to train the model. The S:2 parametrization used every other window (32 windows/reaction). The S:4 parametrization used every fourth window (16 windows/reaction). The S:8 parametrization used 8 windows/reaction. The reference curve is the average of the 4 surfaces. Parts a–f and g–l are the reactions involving the mNuc and Nuc nucleophiles, respectively.

**Figure 4:**
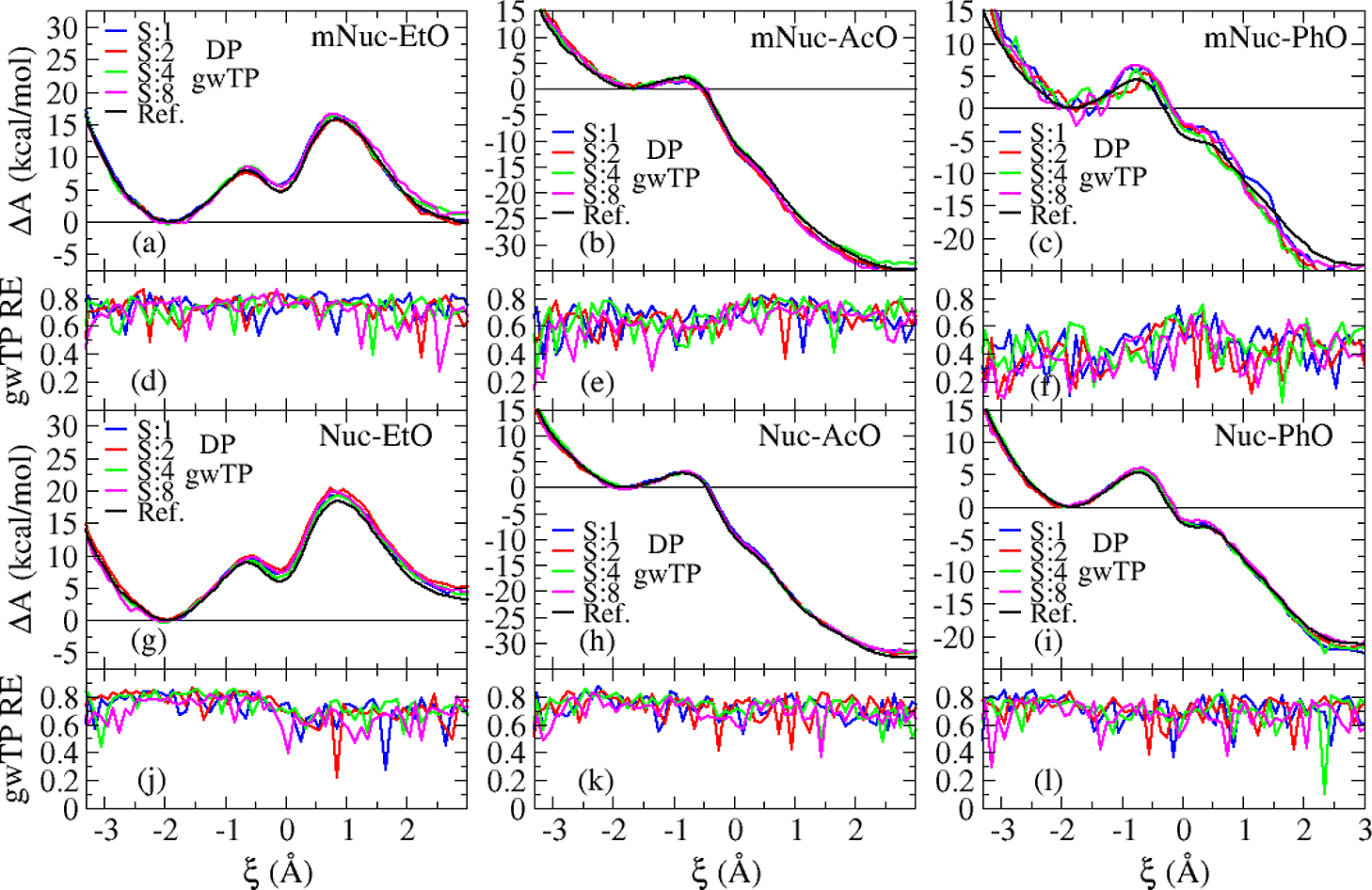
Estimates of the PBE0/6–31G* free energy profile made from gwTP analysis of several DP parametrizations. The S:1 models were parametrized to the NCH–OEt, NHH-OAc, and NHH-OPh reactions using all 64 umbrella windows. The other S/*n* models were parametrized with fewer samples. *n* is the stride in the ξ series of umbrella windows used to train the model. The S:2 parametrization used every other window (32 windows/reaction). The S:4 parametrization used every fourth window (16 windows/reaction). The S:8 parametrization used 8 windows/reaction. The reference curve is the average of the 4 surfaces. Parts a–f and g–l are the reactions involving the mNuc and Nuc nucleophiles, respectively.

The gwTP–estimated PBE0/6–31G* surfaces obtained from the MACE ΔMLP models (see Figure 3) are not sensitive to the gaps in the training data, and the models serve as excellent reference potentials for all 6 reactions, including the 3 reactions that were not included in the parameter refinement. The large reweighting entropies and the striking agreement between the S:1, S:2, S:4, and S:8 MACE models motivated us to use their average as the reference PBE0/6–31G* surface appearing in the comparisons because the amount of sampling performed with AM1/d + MACE far exceeds what can be reasonably afforded from explicit ab initio QM/MM simulation. In contrast, the DP ΔMLP estimates of the PBE0/6–31G* surfaces (see Figure 4) exhibit lower reweighting entropies and more numerical noise. The reweighting entropies are summarized in Table 1. The DP average reweighting entropies decrease as the gaps in the training samples increase from S:1 to S:8; however, the differences are smaller than their standard deviations. The DP models are transferable to the Nuc-EtO reaction, but less so to the mNuc-AcO and mNuc-PhO reactions which systematically have the lowest reweighting entropies among the 6 reactions. The mNuc-PhO surface, in particular, is poorly reproduced; the 4 models contain significant numerical noise and the reweighting entropies range from 0.39 to 0.46.

**Table 1:** Generalized Weighted Thermodynamic Perturbation Reweighting Entropies for Several Parametrizations (Param.) of the MACE and DP Architectures (Arch.)^a^

		Reaction					
param.	arch.	mNuc-EtO	mNuc-AcO	mNuc-PhO	Nuc-EtO	Nuc-AcO	Nuc-PhO
S1	MACE	0.87 ± 0.03	0.87 ± 0.02	0.84 ± 0.04	0.86 ± 0.03	0.85 ± 0.04	0.83 ± 0.04
	DP	0.74 ± 0.07	0.66 ± 0.11	0.45 ± 0.14	0.72 ± 0.10	0.72 ± 0.08	0.71 ± 0.10
S2	MACE	0.87 ± 0.04	0.87 ± 0.02	0.84 ± 0.03	0.86 ± 0.04	0.86 ± 0.03	0.84 ± 0.04
	DP	0.73 ± 0.08	0.66 ± 0.09	0.40 ± 0.15	0.73 ± 0.10	0.72 ± 0.09	0.71 ± 0.09
S4	MACE	0.87 ± 0.03	0.86 ± 0.04	0.84 ± 0.04	0.86 ± 0.04	0.85 ± 0.04	0.83 ± 0.05
	DP	0.72 ± 0.08	0.66 ± 0.11	0.46 ± 0.13	0.73 ± 0.08	0.72 ± 0.08	0.70 ± 0.11
S8	MACE	0.87 ± 0.04	0.86 ± 0.03	0.84 ± 0.04	0.86 ± 0.04	0.85 ± 0.05	0.83 ± 0.05
	DP	0.70 ± 0.10	0.61 ± 0.11	0.39 ± 0.14	0.68 ± 0.09	0.69 ± 0.08	0.66 ± 0.10

a The reweighting corresponds to the prediction of the PBE0/6-31G* free energy from the AM1/d+ΔMLP sampling produced by 4 network parameter sets. This table summarizes the average and standard deviation of the RE values illustrated in Figures 3 and 4.

The AM1/d + MACE and AM1/d + DP free energy surfaces (as opposed to the PBE0/6–31G* estimated from sample reweighting) can be found in the Supporting Information. In brief, the AM1/d + MACE surfaces are nearly indistinguishable from each other and the PBE0/6–31G* target, whereas the AM1/d + DP surfaces exhibit errors—especially for the reactions not included in the refinement.

Figures 5 and 6 and Table 2 extend the comparisons by examining the transferability of the MACE and DP end-state models. The data used to train these models was limited to the ξ = 3 Å and ξ = −2 Å umbrella samples from each reaction. Figure 5 illustrates the free energies of the AM1/d + ΔMLP models, and Figure 6 compares their use as reference potentials to estimate the PBE0/6–31G* surface from wTP analysis. Each plot contains 4 MACE and 4 DP surfaces corresponding to the 4 end-state parametrizations initiated from different random number seeds. Figure 5 illustrates that there are significant variation between the 4 DP end-state models near ξ ≈ 0—the region furthest from the samples used to train the models. In this region, the DP surfaces qualitatively look more similar to AM1/d than the PBE0/6–31G* target because their rate limiting barriers are similar to AM1/d and their mNuc-EtO and Nuc-EtO profiles do not convincingly exhibit early transition states. When used as reference potentials to estimate the PBE0/6–31G* surface (see Figure 6), the DP models continue to show significant variation near ξ ≈ 0. The numerical noise in their wTP estimates are large, and the reweighting entropies are low (0.38 to 0.42). In comparison, the 4 MACE end-state models in Figure 5 show greater agreement with each other and the PBE0/6–31G* target. Furthermore, the MACE end-state models clearly produce intermediates in the mNuc-EtO and Nuc-EtO profiles; however, the depth of the intermediate is too shallow with respect to the reference profile by 2 to 3 kcal/mol. We found this observation to be surprising because the intermediate state corresponds to a pentacoordinate phosphorus structure; the training data only included structures in which either the P–O2′ or P–O5′ bond was fully broken. When wTP analysis is performed on the MACE sampling (see Figure 6), the errors in the mNuc-EtO and Nuc-EtO intermediate state well depths are reduced to 1.5 kcal/mol. There is good agreement between the 4 wTP MACE estimates and qualitative agreement with ab initio reference, but there is noticeable numerical noise in the predicted surfaces. This is consistent with the mediocre reweighting entropy values that range from 0.63 to 0.70.

**Figure 5:**
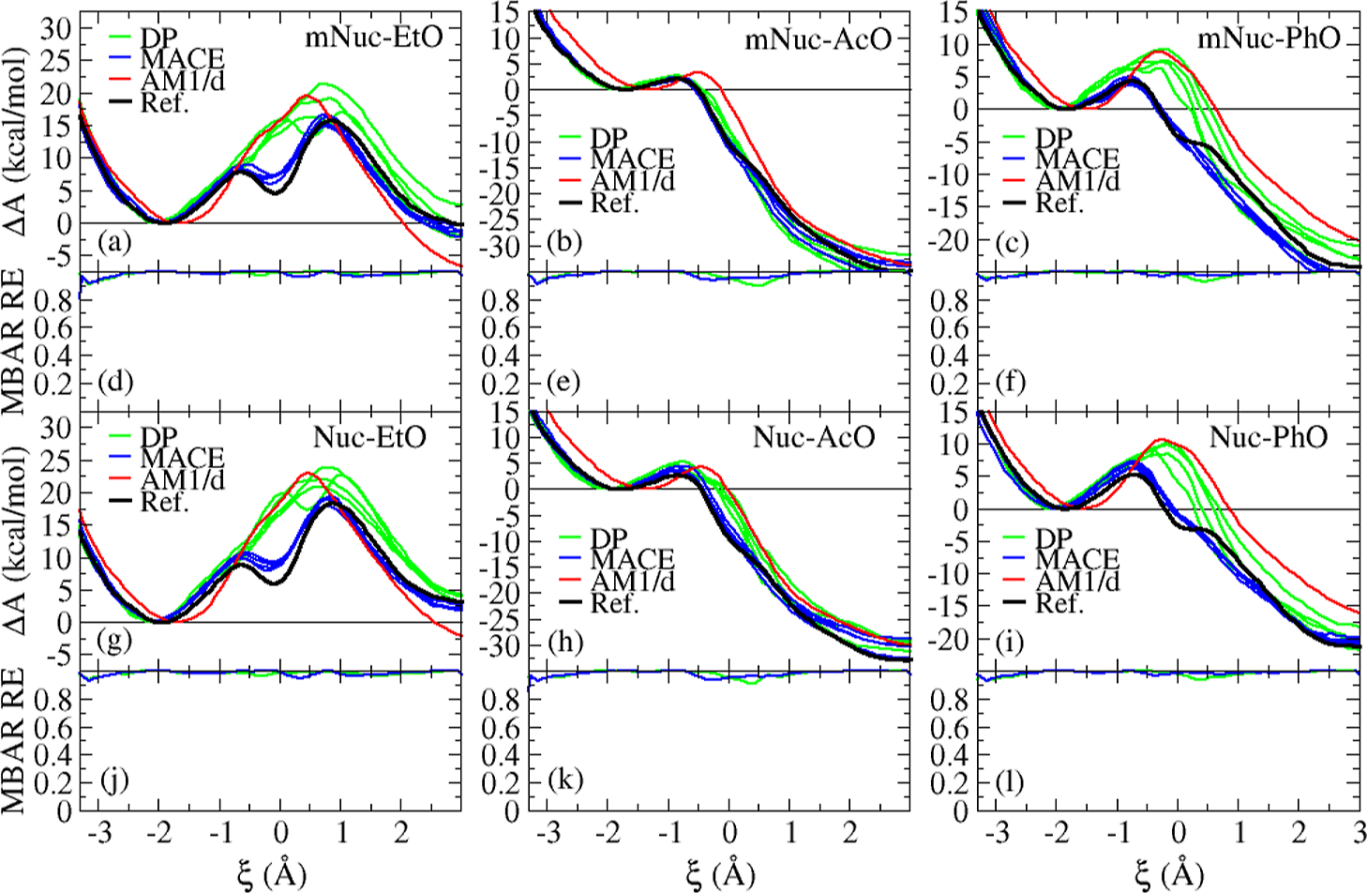
Comparison of AM1/d + ΔMLP free energy profiles, where the ΔMLP is calculated with DP or MACE networks. The ΔMLP corrections were parametrized to the ξ = 3 Å and ξ = – 2 Å umbrella samples of each reaction. Parts a–f and g–l are the reactions involving the mNuc and Nuc nucleophiles, respectively.

**Figure 6:**
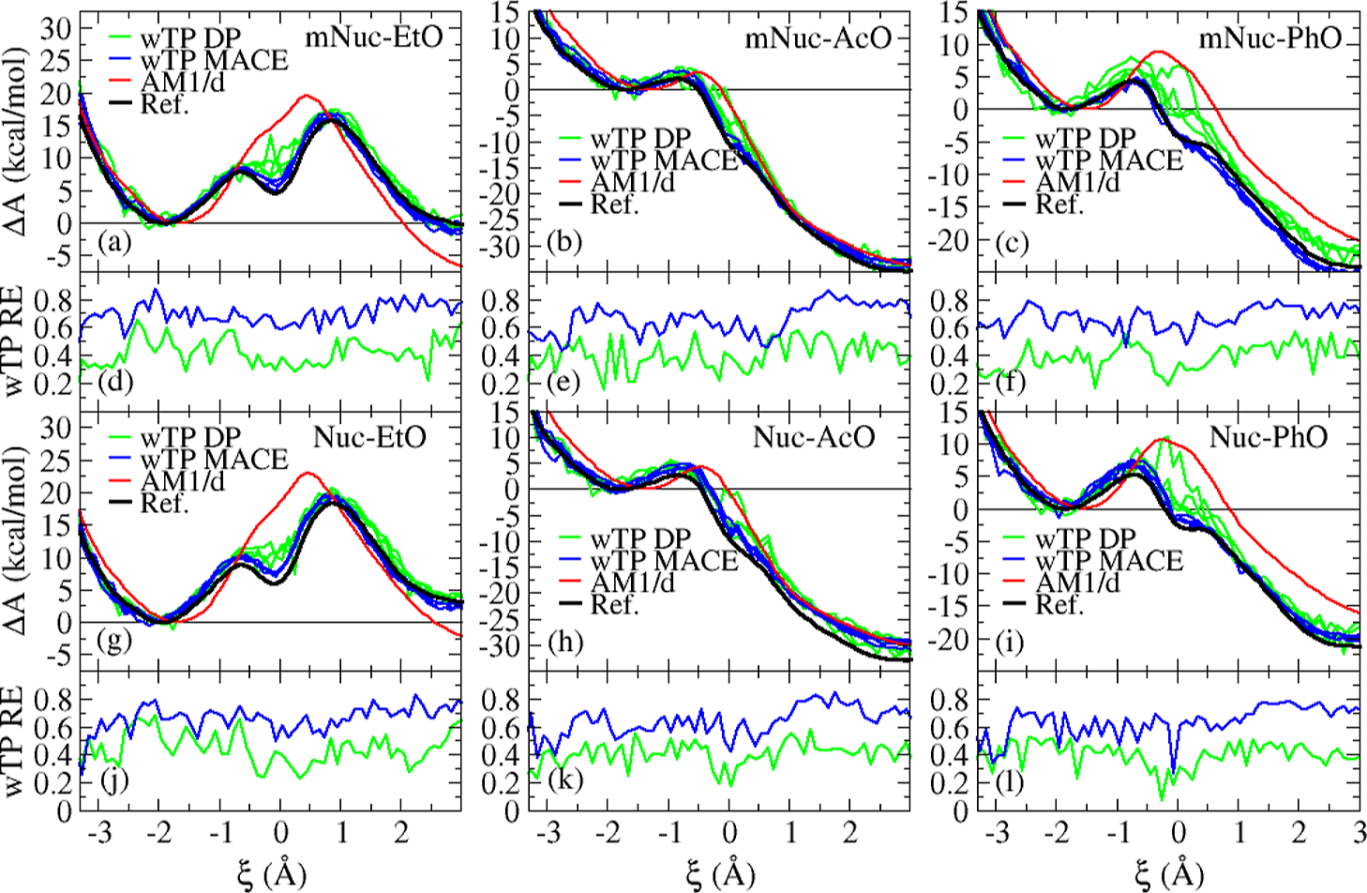
Comparison of PBE0/6–31G* free energy profiles estimated from wTP analysis of the AM1/d + ΔMLP sampling, where the ΔMLP is calculated with DP or MACE networks. The ΔMLP corrections were parametrized to the ξ = 3 Å and ξ = −2 Å umbrella samples of each reaction. Parts a–f and g–l are the reactions involving the mNuc and Nuc nucleophiles, respectively.

**Table 2:** Average Reweighting Entropies of the MACE and DP Architecture (Arch.) End-State Models^a^

		reaction					
weights	arch.	mNuc-EtO	mNuc-AcO	mNuc-PhO	Nuc-EtO	Nuc-AcO	Nuc-PhO
MBAR	MACE	0.98 ± 0.02	0.98 ± 0.02	0.98 ± 0.01	0.98 ± 0.02	0.98 ± 0.02	0.98 ± 0.01
	DP	0.98 ± 0.02	0.98 ± 0.03	0.98 ± 0.02	0.98 ± 0.01	0.98 ± 0.02	0.98 ± 0.02
wTP	MACE	0.70 ± 0.07	0.65 ± 0.11	0.67 ± 0.08	0.66 ± 0.09	0.64 ± 0.10	0.63 ± 0.11
	DP	0.42 ± 0.10	0.40 ± 0.10	0.38 ± 0.10	0.44 ± 0.11	0.40 ± 0.08	0.40 ± 0.09

a The values measure the distribution of the unbiased AM1/d+ΔMLP weights (MBAR) or wTP weights used to estimate the PBE0/6-31G* the free energy. This table summarizes the average and standard deviation of the RE values illustrated in Figures 5 and 6.

One may question if the differences between AM1/d + DP and AM1/d + MACE shown in Figures 5 and 6 are a direct consequence of the ΔMLP energies or a secondary effect caused by structural inconsistencies in the simulations. We have included a comparison of heavy atom coordinate root-mean-square deviations (RMSD) in the Supporting Information to address this possibility. In summary, we performed expensive PBE0/6–31G* QM/MM simulations of the rate limiting transition states to calculate an ensemble averaged ab initio solute structure. The configurations saved from the AM1/d + DP and AM1/d + MACE trajectories were aligned to the ab initio structure to minimize the RMSD. The average and standard deviation of the RMSD values were tabulated, and it was found that the difference between the AM1/d + DP and AM1/d + MACE averages are less than the sum of their standard deviations. This suggests that the observations illustrated Figures 5 and 6 are likely a direct outcome of the ΔMLP corrections rather than a strucutral effect.

### 3.3 Effect of MACE Network Parameters on Accuracy and Performance

Figure 7 and Table 3 compare a series of AM1/d + MACE end-state models. The models differ by varying the network parameters that control the message passing mechanism, such as the equivariant feature maximum angular momentum (*L*), the number of message passing layers (*T*), the correlation order (ν), and the number of channels (*N*). The MACE models in Figures 3, 5, and 6 correspond to the hyperparameters *L* = 1, *T* = 2, ν = 3, *N* = 128. The variations shown in Figure 7 independently adjust each hyperparameter to reduce the complexity of the network. The free energy surfaces produced by these models are largely invariant to adjustment of the network parameters. There is a slight decrease in the PBE0/6–31G* wTP reweighting entropy averages when *L* is reduced from 1 to 0 or *T* is reduced from 2 to 1; however, these differences are smaller than the standard deviation. The reweighting entropies are most sensitive to a reduction of the correlation order from 3 to 1, which causes the entropies to decrease from 0.65 to 0.43.

**Figure 7:**
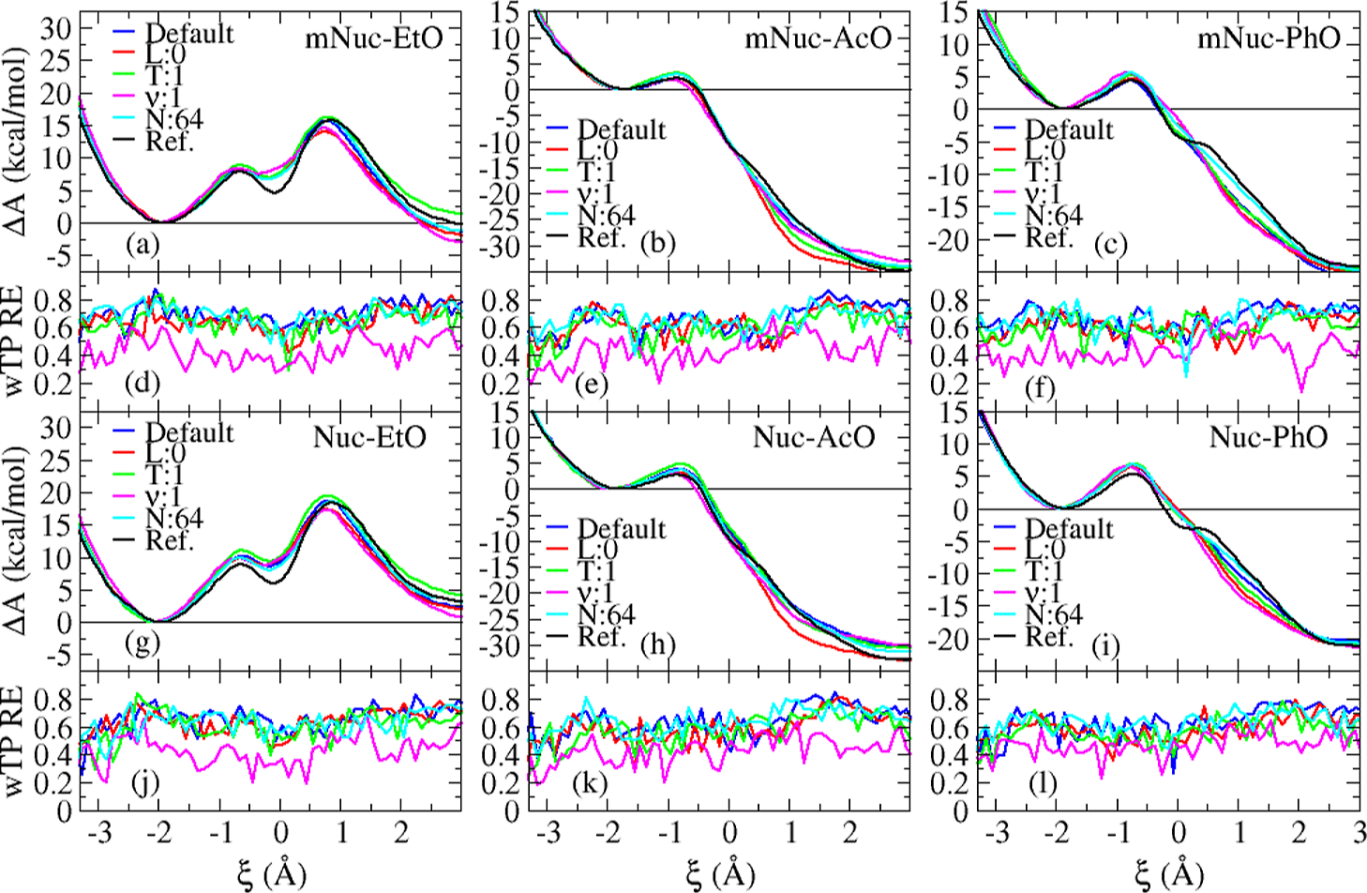
Comparison of AM1/d + MACE end-state model free energy profiles. The reweighting entropies analyze the wTP estimate of the PBE0/6–31G* distributions. The default model hyperparameters are *L* = 1, *T* = 2, ν = 3, and *N* = 128. The other variations differ by reducing the value of a hyperparameter, as indicated in the legend. Parts a–f and g–l are the reactions involving the mNuc and Nuc nucleophiles, respectively.

**Table 3:** Average Reweighting Entropies of Several AM1/d + MACE End-State Models Calculated From PBE0/6-31G* Target Potential wTP Analysis. *L*, *T*, ν, and *N* are the Equivariant Feature Maximum Angular Momentum, the Number of Message Passing Layers, the Correlation Order, and the Number of Channels, Respectively^a^

				reaction					
*L*	*T*	ν	*N*	mNuc-EtO	mNuc-AcO	mNuc-PhO	Nuc-EtO	Nuc-AcO	Nuc-PhO
1	2	3	128	0.70 ± 0.07	0.65 ± 0.11	0.67 ± 0.08	0.66 ± 0.09	0.64 ± 0.10	0.63 ± 0.11
0	2	3	128	0.64 ± 0.08	0.63 ± 0.10	0.61 ± 0.09	0.64 ± 0.08	0.59 ± 0.10	0.59 ± 0.08
1	1	3	128	0.64 ± 0.08	0.60 ± 0.10	0.60 ± 0.08	0.60 ± 0.10	0.57 ± 0.11	0.57 ± 0.09
1	2	1	128	0.43 ± 0.09	0.43 ± 0.11	0.43 ± 0.10	0.43 ± 0.11	0.43 ± 0.10	0.47 ± 0.08
1	2	3	64	0.68 ± 0.07	0.66 ± 0.08	0.66 ± 0.09	0.63 ± 0.08	0.64 ± 0.09	0.63 ± 0.07

a This table summarizes the average and standard deviation of the RE values illustrated in Figure 7.

**Table 4:** Performance of AM1/d and AM1/d + ΔMLP QM/MM Simulation of the mNuc-AcO Reaction^a^

					time (ms/step)	
model	*L*	*T*	ν	*N*	CPU	CPU + GPU
AM1/d	···	···	···	···	114	···
AM1/d + DP	···	···	···	···	250	124
AM1/d + MACE	1	2	3	128	3277	146
	0	2	3	128	1636	136
	1	1	3	128	1558	135
	1	2	1	128	2334	137
	1	2	3	64	1631	133

a The performance metric is the wall-clock time per molecular dynamics step (ms/step, lower is better) evaluated with a Intel Xeon 8358 2.60 GHz and an NVidia V100. *L*, *T*, ν, and *N* are the equivariant feature maximum angular momentum, the number of message passing layers, the correlation order, and the number of channels, respectively.

The observations made in Figure 7 offers some insight into why the AM1/d + MACE models are more accurate and transferable than AM1/d + DP. The DP ΔMLP correction is a type of feed-forward neural network evaluated with 2-body embedding descriptors, whereas most of the MACE ΔMLP potentials appearing in this work were parametrized with a correlation order of 3 (4-body descriptors). One may question if the increased body–order in the MACE descriptors is the source of the differences between the DP and MACE results. This hypothesis is partially supported by Figure 7, which shows that the quality of the AM1/d + MACE reference potential is reduced when the correction is limited to 2-body descriptors.

Table 4 shows the simulation performance of the mNuc-AcO system measured on a single core of a Intel Xeon 8358 2.60 GHz central processing unit (CPU) using AM1/d, AM1/d + DP, and several AM1/d + MACE QM/MM models. The performance is quantified by the average number of milliseconds needed to complete a molecular dynamics time step (smaller values are better performance). The measurements were repeated using a NVidia V100 graphics processing unit (GPU) to evaluate the ΔMLP component of the energy. When calculated on a CPU, the AM1/d + DP method is twice as slow as a standard AM1/d QM/MM potential; however, the AM1/d + MACE models are 14 to 29 times more expensive which makes them impractical to use. Furthermore, reducing the values of MACE network hyperparameters has a significant effect on the model’s CPU performance. Reducing the values of *L* or *T* causes the CPU performance to double. When the GPU is used as a coprocessor to evaluate the ΔMLP, the AM1/d + DP method is only 8% slower than AM1/d QM/MM. The AM1/d + MACE models achieve a similar performance; they are 18% to 28% slower than AM1/d QM/MM. The large difference in CPU performance is likely the consequence of how the two MLP architectures are implemented. The DP architecture is implemented with the TensorFlow libraries, whereas the MACE method is based on PyTorch, whose performance is tuned for GPUs. The AM1/d + MACE GPU timings are not as sensitive to changes in the model hyperparameters as the CPU timings. This could be a consequence of the small size of this application; the GPU calculation may be bottle-necked by memory transfers rather than floating point evaluation.

## 4 Conclusions

We described the adaptation of the MACE message passing neural network for use as a range corrected ΔMLP to improve semiempirical QM and QM/MM interactions. The key modifications include: changing the graph network to avoid direct communication between pairs of MM atoms, forcing the isolated atom MM energy corrections to be zero, and allowing MM and QM atoms of the same element to be trained with different neural network parameters. These changes ensure that the energy is conserved when MM atoms enter or leave the vicinity of the QM region during the course of dynamics.

We trained a series of AM1/d + ΔMLP models to reproduce PBE0/6–31G* QM/MM energies and forces. The models were used to investigate the transferability of the MACE corrections in umbrella sampling free energy applications of 6 nonenzymatic reactions that mimic the RNA transphosphorylation mechanism. We repeated the training and QM/MM umbrella sampling to make comparison with range corrected DeepPot-SE deep neural network ΔMLP models, referred to as AM1/d + DP. We were able to train the different architectures in a consistent manner by utilizing the graph neural network software plugin infrastructure recently incorporated into the DeePMD-kit package. This allowed us to parametrize the methods using the algorithms and optimization hyperparameters interfaced through the DP-GEN software,^64^ which also provides an easy to use framework for query–by–committee active learning.^124,125^

We parametrized “end-state” models that were trained to the reactant and product state structures from all 6 reactions, and these models were refined several times by including varying amounts of umbrella sampling from 3 of the 6 reactions. When the refined models were applied as reference potentials to estimate the PBE0/6–31G* surfaces of all 6 reactions, the AM1/d + MACE method was found to consistently well-reproduce the target surfaces, whereas the AM1/d + DP models exhibited substantially larger numerical noise—especially for those reactions not included in the refinement training. The free energy surfaces were also calculated using several end-state models that differed only in the random number seed used to initiate the neural network parameter optimization. There were large variations in the AM1/d + DP models near the transition states and the surfaces showed greater resemblance to the AM1/d base model than the PBE0/6–31G* target because their barriers were too large and they failed to reliably produce the expected pentacoordinated phosphorus intermediate states. When used as a reference potential, the target surfaces estimated from the AM1/d + DP sampling suffered from numerical noise and low reweighting entropies indicating that the accuracy of the ΔMLP correction is insufficient. In contrast, the AM1/d + MACE surfaces agreed reasonably well with each other and PBE0/6–31G*. The AM1/d + MACE models correctly predicted stable pentacoordinated phosphorus intermediate states even though the training did not include structures with a similar bonding pattern. When used as a reference potential, the AM1/d + MACE models produced less numerical noise and larger reweighting entropies than the AM1/d + DP models.

Several AM1/d + MACE end-state models were compared to investigate the effect of the message passing hyperparameters on the model’s accuracy and performance. Individually reducing the equivariant feature maximum angular momentum and the number of message passing layers had a minimal impact on the free energy surfaces. The largest effect occurred by reducing the correlation order from 3 to 1 which decreased the wTP reweighting entropies from 0.65 to 0.43. The PyTorch implementation of the MACE potential is not well-optimized for inference on CPUs. The AM1/d + MACE performance is 2900% slower than a standard AM1/d QM/MM calculation when performed on a CPU; however, when the ΔMLP is evaluated with a NVidia V100 GPU, the AM1/d + MACE performance is only 28% slower. This performance penalty is only slightly worse than the 8% performance penalty observed with the AM1/d + DP models.

In conclusion, the MACE architecture shows potential as a ΔMLP that may be more transferable than deep neural network models. At the present time, it is not feasible to perform MACE ΔMLP simulations on CPUs; however, its GPU–accelerated performance is sufficiently fast to obtain the sampling needed to calculate free energy surfaces of chemical reactions.

## Supplementary Material



## Notes

Figures that illustrate the smoothness of the QM and QM/MM MACE corrections, the conservation of energy within AM1/d + MACE NVE simulations, energy surfaces analyzed from the AM1/d + MACE and AM1/d + DP S/*n* refined models, and geometrical comparisons (PDF)
