# Supplementary material for: Transferability of MACE Graph Neural Network for Range Corrected Δ‑Machine Learning Potential QM/MM Applications
Source: J Phys Chem B. 2025 May 26;129(22):5477–90. doi: 10.1021/acs.jpcb.5c02006 (PMC12333372; doi:10.1021/acs.jpcb.5c02006)
Supplement: Supplementary file 1 [file jp5c02006_si_001.pdf]

Supporting information for: Transferability of MACE Graph Neural Network for Range Corrected Δ-Machine Learning Potential QM/MM Applications

Timothy J. Giese,[†] Jinzhe Zeng,[‡] and Darrin M. York[*,†]

Laboratory for Biomolecular Simulation Research, Institute for Quantitative Biomedicine and Department of Chemistry and Chemical Biology, Rutgers University, Piscataway, NJ 08854, USA, and School of Artificial Intelligence and Data Science, University of Science and Technology of China, Hefei 230026, China; Suzhou Institute for Advanced Research, University of Science and Technology of China, Suzhou 215123, China; Suzhou Big Data & AI Research and Engineering Center, Suzhou 215123, China

[*]To whom correspondence should be addressed

[†]Laboratory for Biomolecular Simulation Research, Institute for Quantitative Biomedicine and Department of Chemistry and Chemical Biology, Rutgers University, Piscataway, NJ 08854, USA

[‡]School of Artificial Intelligence and Data Science, University of Science and Technology of China, Hefei 230026, China; Suzhou Institute for Advanced Research, University of Science and Technology of China, Suzhou 215123, China; Suzhou Big Data & AI Research and Engineering Center, Suzhou 215123, China

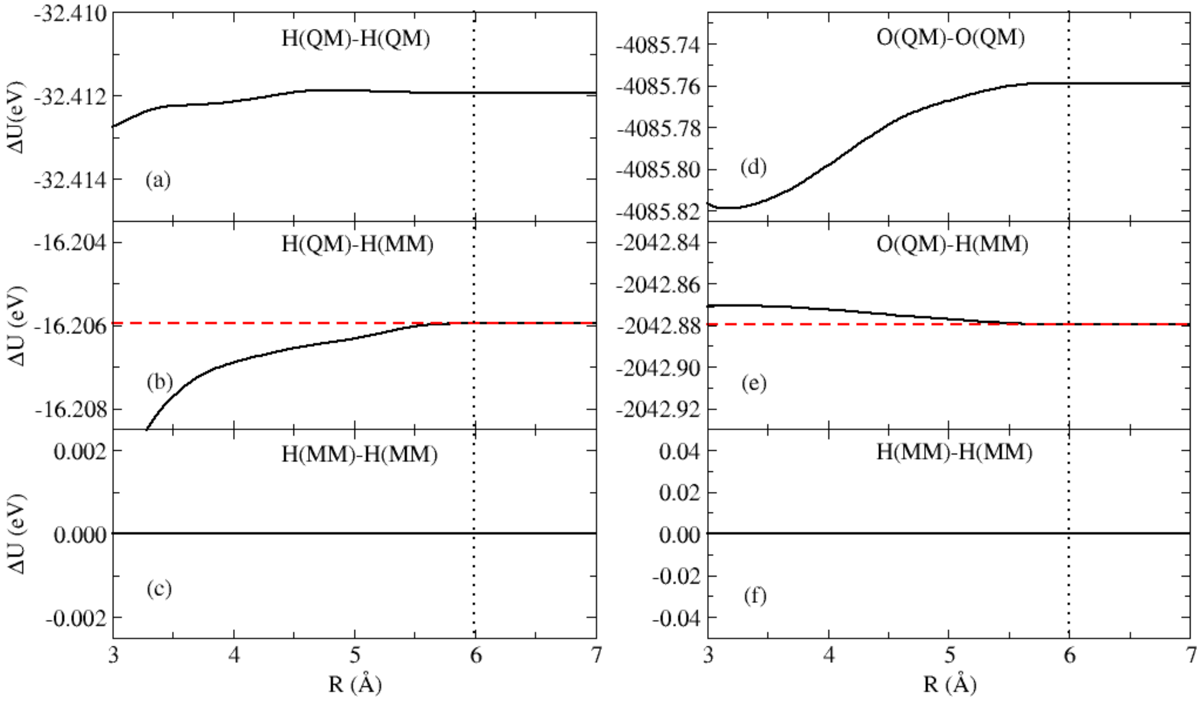

Figure S1: The MACE ΔMLP correction energy of isolated dimers H(QM)-H(QM), H(QM)-H(MM), O(QM)-O(QM), O(QM)-H(MM), and H(MM)-H(MM) as a function of separation using the S:1 AM1/d+MACE parametrization. The vertical dotted lines mark the ΔMLP cutoff. The red dashed lines in parts b and e are the atomic site potentials of H(QM) and O(QM), respectively.

The ΔMLP corrected QM/MM potential is smooth and continuous

Figure S1 illustrates several key properties of the range corrected ΔMLP approach. First, the QM corrections (see Figure S1a and Figure S1d) approach a constant at the ΔMLP cutoff value. The value of the constant is the sum of site energy corrections. Specifically, the constant in Figure S1a is twice the site correction energy of a QM hydrogen, and the limiting value in Figure S1b is twice the site correction energy of QM oxygen. Second, the MM corrections (see Figure S1c and Figure S1f) are zero. Only the QM and QM/MM interactions are corrected. Third, the QM/MM interactions smoothly approach the QM site energy as the MM atom approaches the cutoff. The red dashed lines in Figure S1b and S1e are the site energies of the QM hydrogen and oxygen, respectively.

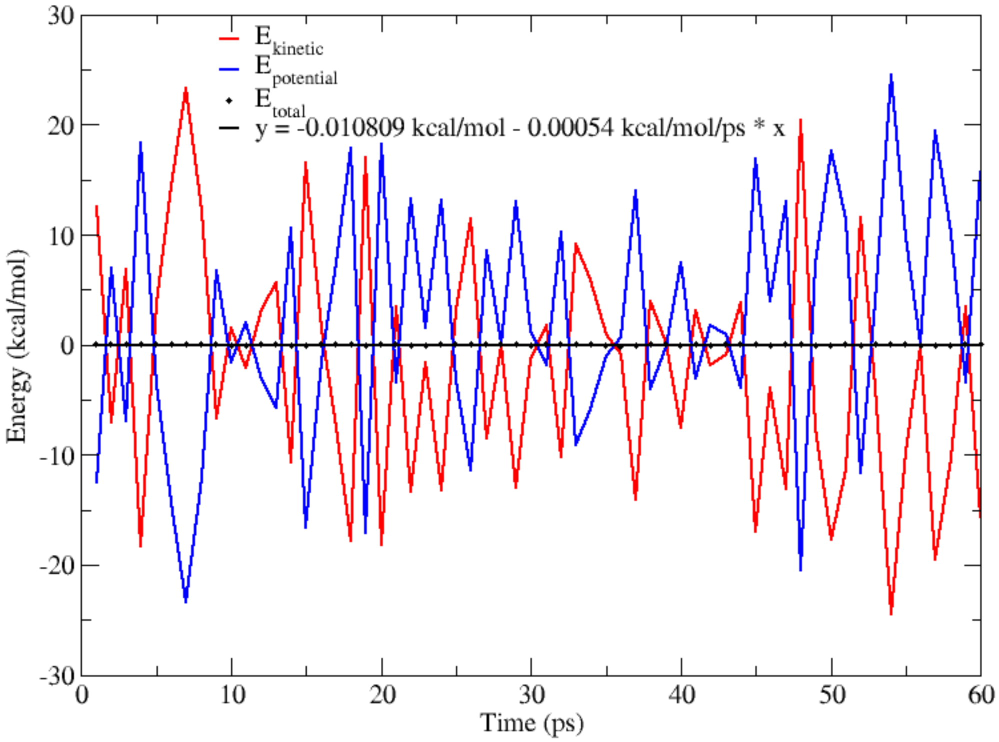

Figure S2: AM1/d+MACE energy time-series of the mNuc-EtO reactant state simulated in the microcanonical ensemble. The plotted values are the kinetic, potential, and total energy averaged over 1 ps intervals. The values have been shifted such that the first time step defines the zero of energy. The black line is a linear regression of the total energies.

Figure S2 demonstrates that the AM1/d+MACE method conserves total energy during simulation in the microcanonical ensemble. Energy conservation is achieved when the potential energy is smooth and its gradient agrees with the analytic forces. The simulation was performed with the S:1 parametrization and a 1 fs time step while being subjected to a harmonic biasing potential centered at $\xi = -2$ Å. The kinetic, potential, and total energies were recorded every step and subsequently averaged over nonoverlapping 1 ps blocks. A linear regression of the total energies produces a slope of 0.00054 kcal/mol/ps, which is consistent with similar energy conservation tests.[S1–S3]

ΔMLP corrected QM/MM free energy profiles

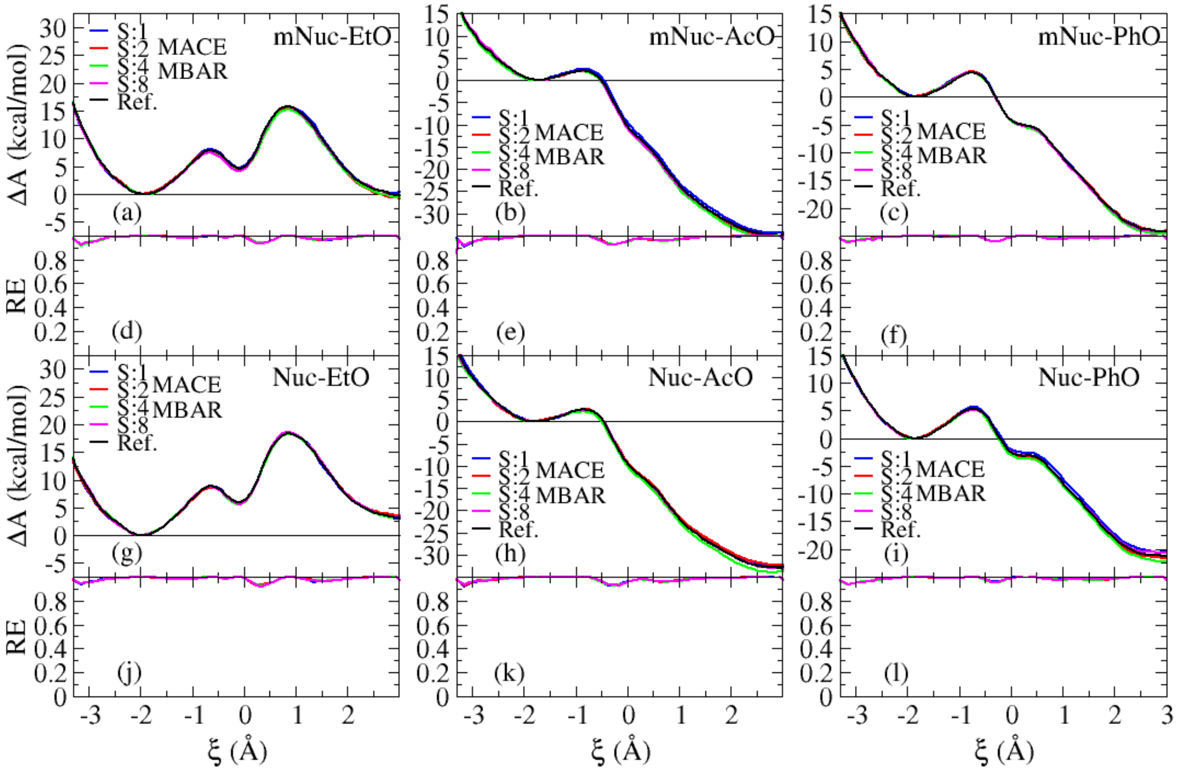

Figure S3: Free energy profiles of several AM1/d+MACE parametrizations. The S:1 models were parametrized to the mNuc-EtO, Nuc-AcO, and Nuc-PhO reactions using all 64 umbrella windows. The other S:*n* models were parametrized with fewer samples. The *n* represents the stride in the series of umbrella windows used to train the model. The S:2 parametrization used every other window (32 mindows/reaction). The S:4 parametrization used every 4th window (16 windows/reaction. The S:8 parametrization used 8 windows/reaction. The reference curve is the average of the 4 surfaces.

Figures S3 and S4 show the free energy surfaces produced by the refined AM1/d+MACE and AM1/d+DP models, respectively. These surfaces were calculated from MBAR analysis of the reaction coordinate distributions,[S4] whereas Figures 3 and 4 in the main manuscript illustrate the PBE0/6-31G* surfaces estimated from reweighted samples.[S5] The AM1/d+MACE surfaces shown in Figure S3 are nearly indistinguishable from the PBE0/6-31G* reference, whereas the AM1/d+DP surfaces exhibit differences – especially for the Nuc-EtO, mNuc-AcO, and mNuc-PhO surfaces which were not included in the model refinement.

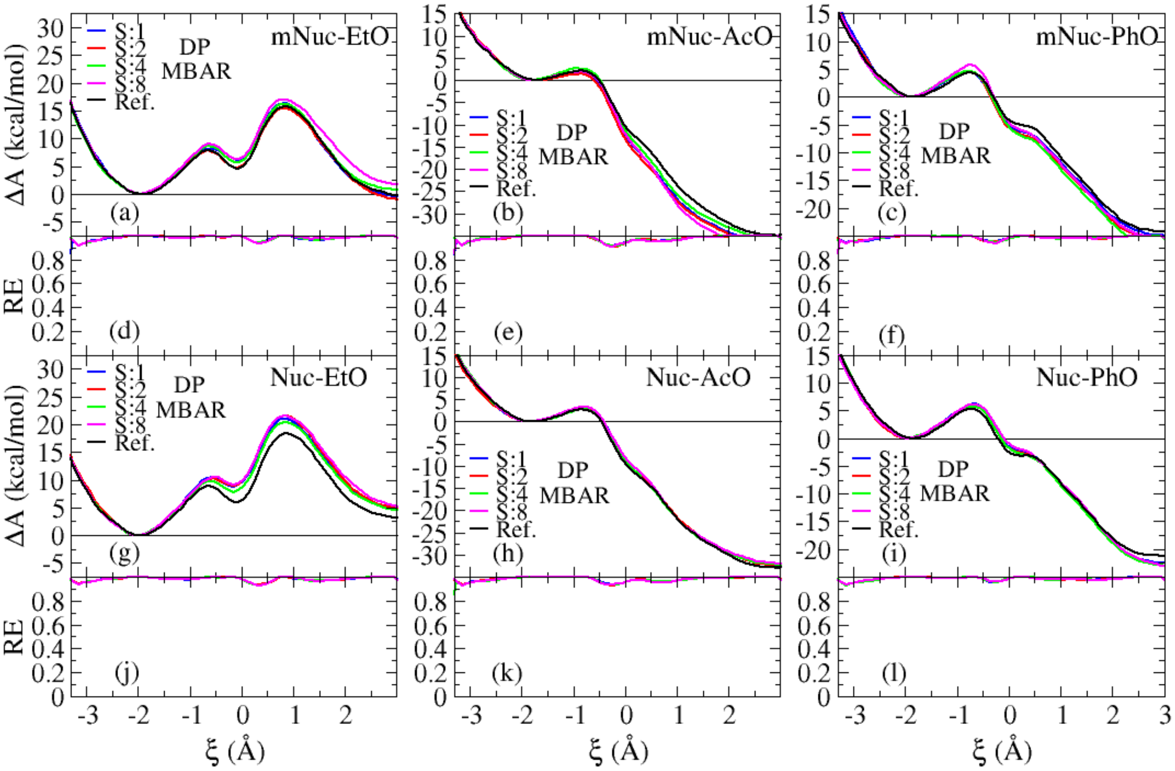

Figure S4: Free energy profiles of several AM1/d+DP parametrizations. The S:1 models were parametrized to the mNuc-EtO, Nuc-AcO, and Nuc-PhO reactions using all 64 umbrella windows. The other S:*n* models were parametrized with fewer samples. The *n* represents the stride in the series of umbrella windows used to train the model. The S:2 parametrization used every other window (32 mindows/reaction). The S:4 parametrization used every 4th window (16 windows/reaction. The S:8 parametrization used 8 windows/reaction. The reference curve is the average of the 4 surfaces.

Comparison of AM1/d+MACE, AM1/d+DP, and PBE0/6-31G* structures

Table S1: Heavy atom coordinate root mean square deviations (Å) of samples near the rate limiting transition state relative to PBE0/6-31G* QM/MM ensemble averaged structures.

Solute	AM1/d+MACE	AM1/d+DP
mNuc-EtO	2.59 ± 0.05	2.54 ± 0.05
mNuc-AcO	2.70 ± 0.03	2.73 ± 0.05
mNuc-PhO	2.64 ± 0.06	2.64 ± 0.04
Nuc-EtO	2.63 ± 0.06	2.62 ± 0.04
Nuc-AcO	2.69 ± 0.06	2.61 ± 0.04
Nuc-PhO	2.66 ± 0.05	2.64 ± 0.05

The main document compared free energy surfaces analyzed from sampling produced by AM1/d+MACE and AM1/d+DP "end–state" models, and it was found that the AM1/d+MACE model produced surfaces that agreed much better to the PBE0/6-31G* reference than AM1/d+DP. One may question if the free energy differences are a direct consequence of the ΔMLP correction or an indirect condequence caused by an underlying structural change. To address this, table S1 compares coordinate root mean square deviations (RMSD) of AM1/d+MACE and AM1/d+DP models relative to PBE0/6-31G* QM/MM sampling. For each method (AM1/d+MACE, AM1/d+DP, and PBE0/6-31G*), we sampled the window closest to the rate limiting transition state. For the EtO, AcO, and PhO leaving groups, we sampled the ξ = 0.90 Å, -0.80 Å, and -0.70 Å windows, respectively. Four initial structures of each window were extracted from the AM1/d+MACE production simulations obtained using the 4 end–state parametrization. Each structure was equilibrated at constant temperature for an additional 10 ps. This was followed by an additional 50 ps of production sampling from which we collected 250 frames. This was repeated for each of the 4 starting structures, such that 1000 frames were collected with each method. An average PBE0/6-31G* structure was calculated by root mean square (RMS) fits of each frame to the starting configuration. All solvent and solute hydrogens were removed before the analysis. Furthermore, the extra C2' methyl group in the mNuc nucleophiles, and all leaving group atoms beyond the C5' position were ex-

cluded. In this manner, we analyze the same subset of heavy atoms in each system. The 1000 RMSD values are calculated upon RMS fitting each frame to the average PBE0/6-31G* structure. The table shows the average and standard deviation of the RMSD values.

The differences in the AM1/d+MACE and AM1/d+DP RMSD values are less than (or about the same as) the sum of their standard deviations. This suggests that the structural differences between the methods is not significant, and the large differences in free energies are a direct consequence of the ΔMLP energies.

Table S2: Heavy atom coordinate root mean square deviations (Å) of samples near the rate limiting transition state. The values are calculated relative to an ensemble averaged solute structure taken from either the AM1/d+MACE or AM1/d+DP simulation.

Solute	AM1/d+MACE Reference		AM1/d+DP Reference	
	AM1/d+MACE	AM1/d+DP	AM1/d+MACE	AM1/d+DP
mNuc-EtO	2.59 ± 0.05	2.56 ± 0.04	2.56 ± 0.06	2.50 ± 0.05
mNuc-AcO	2.64 ± 0.04	2.69 ± 0.05	2.68 ± 0.03	2.70 ± 0.04
mNuc-PhO	2.54 ± 0.05	2.51 ± 0.06	2.51 ± 0.05	2.48 ± 0.06
Nuc-EtO	2.64 ± 0.06	2.64 ± 0.04	2.64 ± 0.06	2.63 ± 0.04
Nuc-AcO	2.64 ± 0.07	2.65 ± 0.04	2.65 ± 0.07	2.57 ± 0.03
Nuc-PhO	2.65 ± 0.05	2.64 ± 0.06	2.65 ± 0.06	2.61 ± 0.05

The AM1/d+MACE and AM1/d+DP RMSD values shown in Table S1 are significantly different from the *ab initio* average structure because the atoms thermally fluctuate during the simulation. To emphasize this point, Table S2 compares the RMSD values when the reference geometry is the simulation–averaged structure taken from either AM1/dd+MACE or AM1/d+DP. The RMSD values in Table S2 are very similar to those in Table S1. Furthermore, the AM1/d+MACE RMSD values are 2.6 Å even when the reference structure is taken from the AM1/d+MACE simulation.

References

(S1) Giese, T. J.; Zeng, J.; Lerew, L.; McCarthy, E.; Tao, Y.; Ekesan, Ş.; York, D. M. Software Infrastructure for Next-Generation QM/MM-ΔMLP Force Fields. *J. Phys. Chem. B* **2024**, *128*, 6257–6271.

(S2) Giese, T. J.; Panteva, M. T.; Chen, H.; York, D. M. Multipolar Ewald methods, 1: Theory, accuracy, and performance. *J. Chem. Theory Comput.* **2015**, *11*, 436–450.

(S3) Giese, T. J.; York, D. M. Ambient-Potential Composite Ewald Method for *ab Initio* Quantum Mechanical/Molecular Mechanical Molecular Dynamics Simulation. *J. Chem. Theory Comput.* **2016**, *12*, 2611–2632.

(S4) Shirts, M. R.; Chodera, J. D. Statistically optimal analysis of samples from multiple equilibrium states. *J. Chem. Phys.* **2008**, *129*, 124105.

(S5) Li, P.; Jia, X.; Pan, X.; Shao, Y.; Mei, Y. Accelerated Computation of Free Energy Profile at ab Initio Quantum Mechanical/Molecular Mechanics Accuracy via a Semi-Empirical Reference Potential. I. Weighted Thermodynamics Perturbation. *J. Chem. Theory Comput.* **2018**, *14*, 5583–5596.
